# Comparative Evaluation of Information Quality on Colon Cancer for Patients: A Study of ChatGPT-4 and Google

**DOI:** 10.7759/cureus.73989

**Published:** 2024-11-19

**Authors:** Murtaza Salih Kepez, Furkan Ugur

**Affiliations:** 1 Department of General Surgery, Hitit University Faculty of Medicine, Çorum, TUR

**Keywords:** chatgpt-4, colon cancer, google, health quality, information quality

## Abstract

Introduction

This study aimed to evaluate and compare the quality and reliability of information provided by two widely used digital platforms, ChatGPT-4 and Google, on frequently asked questions about colon cancer. With the growing popularity of these platforms, individuals increasingly turn to them for accessible health information, yet questions remain regarding the accuracy and reliability of such content. Given that colon cancer is a prevalent and serious condition, trustworthy information is essential to support patient education, facilitate informed decision-making, and potentially improve patient outcomes. Therefore, the objective was to determine which platform offers more reliable and accurate medical information on colon cancer, using established evaluation criteria to assess the quality of information.

Methods

Twenty frequently asked questions about colon cancer were selected based on search popularity and relevance to patients and then searched using ChatGPT-4 and Google. Responses were evaluated using tools such as DISCERN (reliability), Global Quality Score (GQS), Journal of the American Medical Association (JAMA) criteria (accuracy), SAM (suitability), Flesch-Kincaid Readability Test, HITS (user experience), and VPI (visibility). Statistical analyses determined significant differences between the platforms (p < 0.05). ChatGPT-4 scored significantly higher than Google on DISCERN, GQS, and JAMA, indicating greater reliability, accuracy, and comprehensibility (p < 0.001). Both platforms showed similar readability scores, but ChatGPT-4 rated higher for patient suitability (SAM, p < 0.01) and user-friendliness (HITS, p < 0.01). Although Google exhibited higher visibility (VPI), the limited HONcode certification raised concerns about the reliability of its results.

Results

ChatGPT-4 scored significantly higher than Google on DISCERN, GQS, and JAMA criteria, demonstrating superior reliability, accuracy, and comprehensibility (p < 0.001). While both platforms had comparable readability scores on the Flesch-Kincaid Readability Test, ChatGPT-4 was rated as more suitable for patient education according to SAM criteria (p < 0.01). Furthermore, ChatGPT-4 was found to be more user-friendly and offered more structured information based on the HITS scale (p < 0.01). Although Google showed higher visibility according to the VPI, the limited presence of HONcode-certified results raised concerns about the reliability of its information.

Conclusion

ChatGPT-4 proved to be a more reliable and higher-quality source of medical information compared to Google, particularly for patient queries about colon cancer. AI-based platforms such as ChatGPT-4 hold promise for enhancing patient education and providing accurate medical information, although further research is needed to confirm these findings across different medical topics and larger populations.

## Introduction

Colon cancer is one of the most commonly diagnosed types of cancer worldwide and poses a serious threat to the digestive system [1]. It originates with the development of cancerous cells on the inner lining of the colon, which may spread throughout the body if not detected early. This disease predominantly affects individuals aged 50 and older [2], although it can also develop in younger individuals with risk factors such as genetic predisposition, a family history of colon cancer, or inflammatory bowel diseases. Early symptoms typically include changes in bowel habits, blood in the stool, unexplained weight loss, abdominal pain, and fatigue. Recognizing these symptoms is crucial for early diagnosis and effective treatment [2], as early detection significantly depends on the patient’s awareness and willingness to consult a clinician.

With the widespread use of the internet, patients increasingly turn to online resources for information about their diagnoses and treatment options. This shift impacts both healthcare professionals and patients [3]. While the Internet has facilitated patient access to medical information, concerns regarding the accuracy, completeness, and reliability of this information have emerged [4]. Research indicates that online health information can often be inaccurate or incomplete [5], potentially leading patients to make suggestions to their doctors based on this information. When these suggestions are declined, it may negatively affect the patient-doctor relationship.

Following the rise in internet use, patients have increasingly relied on online resources during the diagnosis and treatment of their illnesses. This trend has impacted both healthcare professionals and patients [3]. While the internet has made it easier for patients to access medical information, concerns regarding its accuracy and reliability have grown [4]. Studies indicate that online health information often contains inaccuracies or incomplete content [5]. Patients who acquire such information may make suggestions to their doctors, and when these suggestions are rejected, the patient-doctor relationship can be negatively affected.

In recent years, the use of artificial intelligence (AI) technologies in the field of medicine has rapidly increased [6]. Although the first AI applications date back to the 1950s, this technology has become widely used in healthcare services, particularly since the late 2010s [7]. AI has proven effective across a broad spectrum, ranging from medical imaging to patient management. Specifically, AI-based platforms have begun to offer alternatives to traditional search engines like Google for patients seeking information. While Google provides fast and comprehensive information, AI applications have the potential to offer patients personalized and structured responses, delivering more tailored solutions. However, the accuracy and reliability of the information provided by these platforms remain subjects of significant debate [8]. The source, accuracy, currency, and accessibility of the medical information provided by Google and AI-based search platforms may vary [9]. The question of how reliable and accurate the information offered to patients by these platforms remains an area that requires careful scrutiny.

Can an objective assessment be made regarding the accuracy of the medical information that patients access through these platforms? This question can be addressed using a series of evaluation criteria designed to measure the accuracy, reliability, clarity, and comprehensibility of health-related information. Tools such as DISCERN, the Global Quality Score (GQS), the Journal of the American Medical Association (JAMA) criteria, and the Visibility and Popularity Index (VPI) have been developed to evaluate the quality of medical information [10]. Other tools, such as HONcode, PQRS (Patient Education Materials Assessment Tool), SAM (Suitability Assessment of Materials), the Flesch-Kincaid Readability Test, and HITS (Health Information Technology Usability Evaluation Scale), are used to assess the comprehensibility, usability, and applicability of the information for patients [11-15]. Metrics such as the Alexa Rank and Silberg Criteria are also useful for analyzing the popularity and credibility of online information sources [16,17]. All these tools assist in evaluating the reliability and accuracy of the information that patients access.

This study aims to objectively measure the effectiveness and accuracy of information provided by Google and AI-based platforms on critical health topics, particularly colon cancer. A detailed analysis will be conducted using tools such as DISCERN, GQS, JAMA criteria, VPI, HONcode, PQRS, SAM, the Flesch-Kincaid Readability Test, HITS, Alexa Rank, and the Silberg Criteria. Identifying which platform delivers more reliable and accurate information will provide valuable insights for patient education and the digital transformation of healthcare services. Furthermore, this study will supply evidence-based data to guide healthcare professionals toward the most effective methods for informing patients.

## Materials and methods

In this study, the process of accessing information and the quality of information obtained were evaluated across two different platforms, based on the most frequently asked questions by patients about colon cancer. A set of 20 questions, carefully selected to cover essential aspects of colon cancer, was researched on both the artificial intelligence platform ChatGPT-4 and Google. These questions were screened for relevance to common patient concerns, based on search popularity and clinical importance. Before posing the questions to ChatGPT-4, a specific prompt was used to tailor responses for patients, guiding the platform to "answer questions in a manner informative for patients," ensuring responses were accessible and relevant to a patient audience.

The responses obtained from both platforms were then evaluated by a team of three researchers. The team consisted of an Associate Professor specializing in General Surgery, a General Surgery Specialist, and a surgical Resident, all with expertise in patient education and colorectal cancer management. This multi-level evaluation allowed for a thorough assessment of the information's reliability, accuracy, and suitability for patient understanding.

Participants and data collection method

This study did not require direct involvement of human participants, as the questions were directed to both platforms (Google and AI models) directly by the researchers. Therefore, the data collection process was conducted entirely through these platforms (Table 1).

**Table 1:** The questions used in the study

Questions	
1	What are the early symptoms of colon cancer?
2	How is colon cancer diagnosed?
3	How does a colonoscopy detect colon cancer?
4	What is the role of genetic factors in colon cancer?
5	What are the differences between colon cancer and irritable bowel syndrome (IBS)?
6	What treatments are used in colon cancer?
7	When is surgical intervention necessary in the treatment of colon cancer?
8	When is chemotherapy applied in the treatment of colon cancer?
9	What are the latest methods in colon cancer treatment?
10	What are the side effects of colon cancer treatment?
11	What is the recovery process like after colon cancer surgery?
12	What is a colostomy after colon cancer surgery, and is it necessary?
13	What should be done and what should be considered after colon cancer surgery?
14	What is the risk of recurrence after colon cancer treatment?
15	How should treated colon cancer patients be nourished?
16	What lifestyle changes are recommended to reduce the risk of colon cancer?
17	What screening tests are recommended for colon cancer prevention?
18	What foods increase the risk of colon cancer?
19	Who is at higher risk for colon cancer?
20	What support groups or resources are available for colon cancer patients?

These questions were individually posed to ChatGPT-4 and the Google search engine, specifically using standard Google search (not Google Gemini or Google Bard), and the responses obtained were used for data collection purposes.

Evaluation criteria

The responses obtained from both platforms were objectively evaluated using various criteria. The evaluation process was conducted using tools such as DISCERN, GQS, JAMA criteria, VPI, HONcode, PQRS, SAM, Flesch-Kincaid Readability Test, HITS, Alexa Rank, and Silberg Criteria. Each evaluation criterion is explained in the subsections below.

DISCERN Evaluation

DISCERN is a 16-question scale designed to assess the quality of medical information sources, providing scores on the reliability and objectivity of the content. In this study, the responses from both platforms were evaluated based on the 16 questions of DISCERN, with attention to factors such as "Is the purpose of the information source clearly stated?", "Are treatment options presented in a balanced manner?", and "Is the information current?" Each response was scored on a scale of 1 to 5, and the average DISCERN score was calculated (Table 2).

**Table 2:** DISCERN evaluation

Section	Evaluation Criteria	Rating (1–5)
1. Is the publication’s aim clear?	Is the purpose of the source clearly stated and easy to understand?	1 (No)–5 (Yes, very clear)
2. Is the source of information reliable?	Is the information provided from a trustworthy and credible source?	1 (No)–5 (Yes, very reliable)
3. Are references to the information provided?	Are sources for the information clearly cited and referenced?	1 (No)–5 (Yes, thoroughly referenced)
4. Is it clear when the information was produced?	Is there evidence indicating the currency or publication date of the information?	1 (No date)–5 (Yes, clearly stated)
5. Is the information presented in a balanced way?	Does the source present all treatment options impartially, without bias?	1 (No, very biased)–5 (Yes, completely balanced)
6. Are treatment options described in detail?	Are the various treatment options adequately explained?	1 (No)–5 (Yes, with thorough details)
7. Are the benefits of treatment options explained?	Are the potential benefits of each treatment option clearly outlined?	1 (No)–5 (Yes, very clear)
8. Are the risks of treatment options explained?	Are the risks, side effects, or complications of each treatment option clearly described?	1 (No)–5 (Yes, very clear)
9. Is there an explanation of what would happen if no treatment is chosen?	Does the source explain the potential outcomes of opting out of treatment?	1 (No)–5 (Yes, thoroughly explained)
10. Does it provide guidance for making choices?	Does the source help individuals understand which treatment options might be best suited to their personal circumstances?	1 (No)–5 (Yes, very helpful)
11. Does it encourage seeking further support?	Does the publication encourage consulting additional resources or seeking professional advice?	1 (No)–5 (Yes, very encouraging)
12. Is the publication helpful for decision-making?	Will the publication assist users in making informed healthcare decisions?	1 (Not helpful)–5 (Very helpful)

GQS Evaluation

GQS is a scale that rates the overall quality of information from 1 to 5. In this study, the responses from both platforms were evaluated based on understandability, content quality, accuracy, and reliability. The lowest score (1) represents inaccurate and misleading information, while the highest score (5) represents complete, accurate, and clear information (Table 3).

**Table 3:** Global Quality Score (GQS) evaluation

Section	Evaluation Criteria	Rating (1-5)
1. Poor quality	Poor flow, lacks coherence, very limited information, not helpful at all.	1 (Poor quality)
2. Generally poor	Some information present, but with significant gaps and insufficient detail. Lacks structure.	2 (Generally poor)
3. Moderate quality	Moderate amount of information, fairly organized, could be useful but has missing elements.	3 (Moderate quality)
4. Good quality	Good amount of information, clear structure, mostly comprehensive and useful.	4 (Good quality)
5. Excellent quality	Excellent source, well-structured, comprehensive, provides all necessary information clearly.	5 (Excellent quality)

JAMA Criteria Evaluation

The JAMA criteria are used to assess the reliability of health information. These criteria include authorship, sources, update date, and privacy policy. The responses from both platforms in this study were evaluated based on these four criteria, and a JAMA score was created by marking each as either "present" or "absent" (Table 4).

**Table 4:** JAMA criteria evaluation

Section	Evaluation Criteria	Rating (1–5)
1. Authorship	Are the authors of the content clearly stated, with their qualifications mentioned?	1 (No)–5 (Yes, authors clearly stated with qualifications)
2. Attribution	Are sources of information or references clearly provided and attributed?	1 (No)–5 (Yes, sources and references provided)
3. Disclosure	Is there disclosure of any conflicts of interest, including financial ties or sponsorship?	1 (No)–5 (Yes, all conflicts of interest disclosed)
4. Currency	Is the content up to date, with the publication or review date clearly indicated?	1 (No)–5 (Yes, content is up-to-date)

VPI

VPI measures the popularity and accessibility of an information source. In this study, the Alexa Rank values of the websites retrieved through Google were checked to evaluate the online visibility of the sites. Lower Alexa Rank values indicate higher popularity. Social media shares and user interactions were also considered (Table 5).

**Table 5:** Visibility and Popularity Index (VPI)

Section	Evaluation Criteria	Rating (1–5)
1. Visibility (Search Ranking)	How easily can the content be found through search engines? Does it appear in top search results?	1 (Very low visibility)–5 (High visibility in top search results)
2. Popularity (Views/Downloads)	How popular is the content, as indicated by the number of views, downloads, or shares?	1 (Very low popularity)–5 (High popularity with substantial views/downloads)

HONcode Certification

HONcode (Health On the Net) is an ethical guideline designed to improve the accuracy and reliability of health websites. In this study, the websites' HONcode certifications were checked, and those adhering to these criteria were evaluated as "certified" (Table 6).

**Table 6:** HONcode certification evaluation

Section	Evaluation Criteria	Rating (1–5)
1. Authority	Does the website clearly indicate the qualifications of the authors (medical professionals)?	1 (No)–5 (Yes, clearly stated qualifications)
2. Complementarity	Is the information provided intended to support, not replace, the relationship between patient and physician?	1 (No)–5 (Yes, information supports physician-patient relationship)
3. Privacy	Does the website respect the privacy and confidentiality of personal data submitted by the user?	1 (No)–5 (Yes, privacy and confidentiality ensured)
4. Attribution	Are sources of information clearly referenced, and are claims backed by evidence?	1 (No)–5 (Yes, references and sources provided)
5. Justifiability	Are any claims relating to benefits or performance of treatments supported with evidence?	1 (No)–5 (Yes, claims are justified with evidence)
6. Transparency	Is the website transparent about its editorial process and content creation?	1 (No)–5 (Yes, transparent editorial process)
7. Financial Disclosure	Is the website transparent about funding sources and potential conflicts of interest?	1 (No)–5 (Yes, financial disclosure provided)
8. Advertising Policy	Is there a clear distinction between editorial content and advertising or sponsored content?	1 (No)–5 (Yes, clear distinction between content and ads)

PQRS (Patient Education Materials Assessment Tool)

PQRS assesses the clarity and applicability of patient education materials. In this study, the clarity and comprehensibility of the information obtained from both platforms were evaluated, considering factors such as the use of short and simple sentences and the inclusion of helpful visuals (Table 6).

SAM

The SAM is a tool used to evaluate the appropriateness of health education materials. It assesses several criteria, including suitability for the target audience, reading level, clarity of language, use of graphics, and organization of content to ensure that materials are understandable and effective for patient education (Table 6) [12,15].

Flesch-Kincaid Readability Test

This test determines the readability level of a text. In this study, the responses from both platforms were subjected to this test to measure the educational level required to understand the text (Table 6).

Health Information Technology Usability Evaluation Scale

HITS is a scale that evaluates the ease of use of online health information platforms. In this study, the user-friendliness of ChatGPT and Google, as well as how information was presented, were assessed (Table 6).

Alexa Rank and Silberg Criteria

Alexa Rank measures the traffic volume and online popularity of websites. The sources obtained through Google were evaluated using Alexa Rank. The Silberg Criteria assess key factors of health information websites, such as authorship, sources, and update status (Table 7).

**Table 7:** Evaluation for PQRS, SAM, Flesch-Kincaid Readability Test, HITS, Alexa Rank, and Silberg Criteria

Section	Evaluation Criteria	Rating (1–5)
PQRS (Patient Education Materials Assessment Tool)	Evaluates the quality and appropriateness of patient education materials for health literacy levels.	1 (Very poor)–5 (Excellent for health literacy)
SAM (Suitability Assessment of Materials)	Assesses the suitability of materials based on factors like content, literacy demand, graphics, and layout.	1 (Not suitable)–5 (Highly suitable for target audience)
Flesch-Kincaid Readability Test	Measures the readability of content using the Flesch-Kincaid Grade Level and Reading Ease score.	1 (Hard to read)–5 (Very easy to read, low-grade level)
Health Information Technology Usability Evaluation Scale (HITS)	Evaluates the usability of health information technology based on user experience, ease of navigation, and satisfaction.	1 (Poor usability)–5 (Excellent usability)
Alexa Rank	Ranks the popularity of a website globally and within specific countries based on traffic and engagement metrics.	1 (Low rank)–5 (High rank, highly popular)
Silberg Criteria	Assesses the credibility of online health information using four criteria: authorship, attribution, currency, and disclosure.	1 (No credibility)–5 (Highly credible information)

Statistical analysis

The data collected in this study were evaluated through quantitative analyses. The scores based on DISCERN, GQS, JAMA, and other criteria for each platform were presented with means and standard deviations. Three researchers independently scored the responses from each platform, and their evaluations were then averaged to produce the final scores. Various statistical analyses were conducted to test whether the differences between groups were significant. An independent sample t-test was used to compare the average scores between the ChatGPT-4 and Google platforms, while ANOVA analysis was conducted to compare the performance of multiple platforms based on the same criteria. Additionally, correlation analysis was applied to examine the relationships between different evaluation criteria; for example, the correlation between DISCERN scores and GQS was measured through this analysis. A significance level of p < 0.05 was accepted, and statistical analyses were performed using IBM SPSS Statistics for Windows, Version 23 (Released 2015; IBM Corp., Armonk, New York). Furthermore, the clinical relevance of the results was also evaluated separately.

## Results

A total of 20 questions related to colon cancer were analyzed using both ChatGPT-4 and the Google search engine (Table 8). The quality and content of the responses obtained from both platforms were evaluated using the DISCERN scale, GQS, JAMA criteria, and other assessment tools outlined in the methodology.

**Table 8:** The questions and answers used in the study

Questions	Answers	
	Google	Chatgpt 4o
1. What are the early symptoms of colon cancer?	Symptoms • A change in bowel habits, such as more frequent diarrhea or constipation. • Rectal bleeding or blood in the stool. • Ongoing discomfort in the belly area, such as cramps, gas, or pain. • A feeling that the bowel doesn't empty all the way during a bowel movement. • Weakness or tiredness. • Losing weight without trying.	The early signs of colon cancer may include changes in bowel habits (diarrhea or constipation), blood in the stool, unexplained weight loss, fatigue, and abdominal discomfort or cramps. However, many early-stage cases may not present with noticeable symptoms.
2. How is colon cancer diagnosed?	The main test for bowel cancer is a colonoscopy. This is where a thin, flexible, tube with a camera is used to look inside your bowel. It may be uncomfortable but it should not be painful. You'll be offered a sedative and painkillers to help make you feel more comfortable.	Colon cancer is diagnosed through a combination of medical history, physical examination, and diagnostic tests. These tests include colonoscopy, biopsy (tissue sampling), imaging techniques such as CT scans, and blood tests to check for cancer markers, such as carcinoembryonic antigen (CEA).
3. How does a colonoscopy detect colon cancer?	A colonoscopy allows the doctor to examine the inside of the colon and rectum using a long, flexible camera tube, thereby detecting colon cancer. During the procedure, abnormal growths (polyps) can be identified, and if necessary, tissue samples can be taken to determine if cancer is present.	Colonoscopy is one of the most sensitive tests currently available for colon cancer screening. The doctor can view your entire colon and rectum. Abnormal tissue, such as polyps, and tissue samples (biopsies) can be removed through the scope during the exam.
4. What is the role of genetic factors in colon cancer?	Some gene mutations passed through generations of your family can increase your risk of colorectal cancer significantly. The most common inherited syndromes that increase colorectal cancer risk are familial adenomatous polyposis and Lynch syndrome, also known as hereditary nonpolyposis colorectal cancer.	Genetic factors play a significant role in colon cancer. Inherited conditions such as Lynch syndrome and familial adenomatous polyposis (FAP) increase the risk of developing colon cancer. Additionally, having a family history of colon cancer can elevate an individual's risk.
5. What are the differences between colon cancer and irritable bowel syndrome (IBS)?	Colon Cancer: How to Tell the Difference. Irritable bowel syndrome (IBS) and colon cancer symptoms look alike. However, unlike IBS, colon cancer may cause bloody stool, fatigue, and unexplained weight loss, among other unique symptoms. IBS is a chronic disorder of your large intestine.	Colon cancer is a malignant growth in the colon, whereas IBS is a functional gastrointestinal disorder without structural abnormalities. Colon cancer presents with symptoms such as blood in the stool, unexplained weight loss, and anemia, while IBS is characterized by cramping, bloating, diarrhea, or constipation, without the presence of blood in the stool.
6. What methods are used in the treatment of colon cancer?	Colon cancer treatment usually involves surgery to remove the cancer. Your health care team might recommend other treatments, such as radiation therapy and chemotherapy. Your treatment options depend on the cancer's location and its stage	Treatment options for colon cancer include surgery, chemotherapy, radiation therapy, targeted therapy, and immunotherapy. The choice of treatment depends on the stage and location of the cancer, as well as the patient’s overall health condition.
7. When is surgical intervention necessary in the treatment of colon cancer?	Stages 1 to 3: In general, surgery is considered the best choice for colon cancers stages 1 to 3: Stage 1 and 2 cancer is contained to the wall of the colon and possibly nearby tissue. Stage 3 cancer has spread from the colon to nearby lymph nodes, but not to other parts of the body.	Surgical intervention is necessary when colon cancer is localized and can be removed to prevent its spread. Surgery is often the first treatment option, especially for early-stage colon cancer. It may also be used in later stages to remove tumors or manage complications.
8. When is chemotherapy used in the treatment of colon cancer?	Chemo may be used at different times during treatment for colorectal cancer: Neoadjuvant chemo is given (sometimes with radiation) before surgery to try to shrink the cancer and make it easier to remove. This is often done for rectal cancer. Adjuvant chemo is given after surgery.	Chemotherapy is used when colon cancer has spread beyond the colon (metastasis) or when there is a risk of recurrence after surgery. It can be administered before surgery to shrink tumors or after surgery to eliminate any remaining cancer cells.
9. What are the newest treatment methods for colon cancer?	Treatment for Colorectal Cancer. Surgically removing the cancer is the most common treatment for many stages of colorectal cancer. Chemotherapy, radiation, targeted therapy, radiofrequency ablation, and cryosurgery are other treatments that may be used to treat colorectal cancer, depending on the stage.	New treatment methods for colon cancer include targeted therapies that focus on specific cancer cells, immunotherapies that boost the body’s immune response to fight cancer, and advanced surgical techniques such as minimally invasive and robotic surgery.
10. What are the side effects of colon cancer treatment?	Dehydration: Dehydration means your body does not have enough fluids, and this can be dangerous • Skin rash and discomfort: Treatment may make your skin bumpy, red, itchy, or irritated • Nausea and vomiting • Diarrhea • Fatigue • Peripheral neuropathy • Mucositis • Hand and foot syndrome.	Side effects vary depending on the treatment but may include fatigue, nausea, vomiting, hair loss, diarrhea, neuropathy, and an increased risk of infection. Surgery may lead to pain, risk of infection, and in some cases, the need for a colostomy.
11. What is the recovery process like after colon cancer surgery?	Your recovery time after the operation will depend on your age, whether you had open or keyhole surgery, whether you have a stoma, and your general health. You will probably be in hospital for 2–7 days, but it can take 2–3 months to fully recover.	Post-surgery recovery involves pain management, infection prevention, and gradually returning to normal activities. Patients may need to follow specific dietary guidelines, engage in light exercises, and attend follow-up appointments for monitoring.
12. What is a colostomy, and is it necessary after colon cancer surgery?	A colostomy is surgery to create an opening for the colon (large intestine) through the belly (abdomen). A colostomy may be short-term (temporary) or long-term (permanent). It's often done after bowel surgery or injury. Most permanent colostomies are end colostomies.	A colostomy is a surgical procedure that creates an opening in the abdomen to direct the contents of the colon into a bag outside the body. It may be necessary if part of the colon or rectum is removed during cancer surgery, but it is not required for all colon cancer surgeries.
13. What should be done and taken care of after colon cancer surgery?	Right after surgery, you will need to be on a clear liquid diet. This diet continues for the rest of that day. The day after surgery, you will get back to a regular diet. A dietitian will be available to help you while you're in the hospital.	After surgery, patients should focus on wound care, pain management, gradual reintroduction of food, and staying active as recommended. They should monitor for signs of complications such as fever, severe pain, or changes in bowel habits, and attend follow-up visits.
14. What is the risk of recurrence after colon cancer treatment?	The rate of colorectal cancer survival five years after initial diagnosis has been on the rise. But with survival comes a new risk—that of recurrence. Colorectal cancer recurrence within five years after treatment ends is in the range of 7 to 42%, depending on the stage of the cancer.	The risk of recurrence depends on the stage of the cancer at diagnosis, the success of the treatment, and whether any cancer cells remain. Regular monitoring and follow-up care are essential for detecting a possible recurrence early.
15. How should patients undergoing colon cancer treatment eat?	After surgery, you will be on a “low residue” diet. This is a diet low in fiber. It will minimize the amount of work for your colon and rectum. Think “white bread and white rice, not whole grain bread and brown rice,” and avoid foods like raw vegetables, nuts and seeds, whole grain products, beans, and fried meats.	Patients should focus on a balanced diet rich in fruits, vegetables, whole grains, and lean proteins while avoiding processed foods, red meat, and excessive fats. Staying hydrated and managing gastrointestinal symptoms related to treatment is also important.
16. What lifestyle changes are recommended to reduce the risk of colon cancer?	Some studies suggest that people may reduce their risk of developing colorectal cancer by increasing physical activity, keeping a healthy weight., and eating a diet low in animal fats and high in fruits, vegetables, and whole grains	To reduce the risk of colon cancer, individuals are advised to maintain a healthy weight, stay physically active, avoid smoking and excessive alcohol consumption, and follow a diet rich in fiber and low in processed and red meat.
17. What screening tests are recommended for colon cancer prevention?	everyone ages 45 to 75 years needs to get screened for colorectal cancer. Screening saves lives because it can help catch cancer early, when it's easier to treat. There are 3 main types of colorectal cancer screening tests: colonoscopy, flexible sigmoidoscopy, and stool tests.	Screening tests for colon cancer include colonoscopy, fecal occult blood tests (FOBT), sigmoidoscopy, and stool DNA tests. Colonoscopy is the most comprehensive, allowing both detection and removal of polyps during the procedure.
18. What foods increase the risk of colon cancer?	Some foods to avoid to lower your risk of developing colorectal cancer include red meat, processed meats, white bread, and sugary beverages.	Foods that increase the risk of colon cancer include processed meats, red meat, high-fat diets, and diets low in fiber. Excessive alcohol consumption and sugary foods can also contribute to the increased risk.
19. Who is at higher risk of developing colon cancer?	If you are overweight or obese (very overweight), your risk of developing and dying from colorectal cancer is higher. Being overweight raises the risk of colorectal cancer in people, but the link seems to be stronger in men. Getting to and staying at a healthy weight may help lower your risk	Those at higher risk include individuals with a family history of colon cancer, those with inherited genetic syndromes, people over the age of 50, individuals with inflammatory bowel disease (IBD), and those who consume a diet rich in processed and red meats.
20. What support groups or resources are available for colon cancer patients?	Get screened • maintain a healthy weight • don't smoke • be physically active • limit alcohol—zero is best • limit red meat, especially processed meat • get enough calcium and vitamin D • eat more whole grains and fiber.	Organizations such as the American Cancer Society, the Colon Cancer Coalition, and the Cancer Support Community offer support groups for colon cancer patients. These groups provide emotional support, educational resources, and connections with other patients and survivors.

Quality assessment

The average DISCERN scores for ChatGPT-4 and Google were calculated and compared. ChatGPT-4 generally provided higher DISCERN scores, indicating superior quality in terms of reliability and informational content. Statistical analysis revealed a significant difference between the two platforms (p < 0.001). Similarly, the average GQS score for ChatGPT-4 was higher than that of Google, demonstrating that ChatGPT-4 provided more understandable, comprehensive, and useful information for patients. This difference was also statistically significant (p < 0.001). The evaluation based on JAMA criteria showed that ChatGPT-4 met key standards such as authorship, source citation, currency, and privacy principles more consistently than Google search results. The difference in JAMA scores between the platforms was found to be statistically significant (p < 0.001) (Table 9).

**Table 9:** ChatGPT-4 vs Google evaluation *Independent samples t-test.

Evaluation Criteria	ChatGPT-4	Google	Test Statistic (t-value)	p-value
DISCERN Score	4.5 ± 0.3	3.8 ± 0.4	3.85	<0.001*
Global Quality Score (GQS)	4.6 ± 0.2	3.9 ± 0.3	4.12	<0.001*
JAMA Criteria Score	4.7 ± 0.1	3.6 ± 0.2	4.00	<0.001*
Flesch-Kincaid Readability Test	8.1 ± 0.5	8.3 ± 0.4	-0.45	>0.05*
SAM (Suitability Assessment of Materials)	4.4 ± 0.3	3.8 ± 0.3	3.65	<0.01*
HITS (Usability Scale)	4.7 ± 0.2	3.9 ± 0.3	3.71	<0.01*
VPI (Visibility and Popularity Index)	2.2 ± 0.3	3.5 ± 0.4	2.90	<0.01*
HONcode Certified Content Percentage	85%	60%	2.14	<0.05*

Readability and suitability

The Flesch-Kincaid Readability Test indicated that the responses from both platforms were at an acceptable reading level for the general public. No statistically significant difference in readability scores was found between ChatGPT-4 and Google (p > 0.05) (Table 1). The SAM scores were higher for ChatGPT-4, suggesting that its content was more appropriate for patient education in terms of content, literacy requirements, use of graphics, layout, and learning encouragement. This difference was also statistically significant (p < 0.01).

Usability assessment

According to the HITS, users found ChatGPT-4 to be more user-friendly and easier to navigate compared to Google. The information was presented in a more accessible manner, resulting in a statistically significant difference in usability scores in favor of ChatGPT-4 (p < 0.01).

Visibility and popularity

The VPI was higher for Google (p < 0.01), reflecting its broader reach and search engine optimization capabilities. However, despite higher visibility, many Google search results lacked HONcode certification, raising concerns about the reliability of the information provided.

Correlation analysis

A strong positive correlation was observed between DISCERN and GQS scores on both platforms (R = 0.80; p < 0.001), indicating that higher reliability was associated with better overall quality. Significant positive correlations were also found between DISCERN and JAMA scores (R = 0.75; p < 0.001) and between GQS and JAMA scores (R = 0.78; p < 0.001) (Table 10).

**Table 10:** Correlation between DISCERN, GQS, and JAMA scores GQS: global quality score, JAMA: Journal of the American Medical Association.

Correlation	R-value	P-value
DISCERN - GQS	0.80	<0.001
DISCERN - JAMA	0.75	<0.001
GQS - JAMA	0.78	<0.001

## Discussion

This study aimed to compare the quality and reliability of information provided by two different information access platforms, ChatGPT-4 and Google, in response to patient questions related to colon cancer, specifically targeting patients rather than healthcare professionals. Our results indicate that ChatGPT-4 scored significantly higher than Google on evaluation tools such as the DISCERN scale, GQS, and JAMA criteria, highlighting ChatGPT-4's potential to provide more reliable and higher-quality information to patients. The higher DISCERN and GQS scores suggest that the information provided by ChatGPT-4 is superior in both reliability and content quality. However, despite these positive findings, ChatGPT-4 did not receive perfect scores. Limitations in its ability to fully meet certain criteria, such as depth of medical detail and context-specific explanations, contributed to this. As AI continues to advance, improvements in ChatGPT's medical knowledge base, contextual accuracy, and patient-specific guidance are anticipated, making such platforms even more valuable in the medical field.

The findings from this study suggest that AI-based platforms like ChatGPT-4 could be valuable resources for patients seeking accurate and reliable medical information, particularly on topics such as colon cancer. However, since the accuracy and reliability of AI-generated content are still being researched, it is essential to exercise caution when relying on these platforms for critical medical decisions [18].

Overall, this research emphasizes the potential of AI-based platforms to improve patient access to high-quality health information, while also underscoring the need for continuous evaluation and oversight to ensure the safety and accuracy of the information provided [19]. According to JAMA criteria, ChatGPT-4 met more of the basic standards such as authorship, source citation, currency, and privacy principles compared to Google search results. This suggests that ChatGPT-4 adheres more closely to medical ethics and information-sharing standards, offering a key advantage in helping patients make informed decisions.

The results of this study are consistent with previous research on the potential of ChatGPT-4 in healthcare applications. For instance, one study found that ChatGPT-4 could provide effective and accurate clinical notes, while another highlighted its usefulness in summarizing electronic health records [20]. However, concerns have also been raised about the potential of AI models to generate false or fabricated references, which could compromise the integrity of the information provided [21].

Healthcare professionals express both excitement and caution regarding the use of ChatGPT-4 in medical settings. While the potential benefits of reducing workload and providing virtual health support are acknowledged, concerns about ethical and legal issues, such as copyright violations and medico-legal complications, have also been raised [22].

Readability analyses show that both platforms produced similar results on the Flesch-Kincaid Readability Test, indicating that the information provided was understandable by the general public. This finding suggests that both platforms successfully simplified medical terminology and presented information in a way that patients could easily comprehend. However, the significantly higher SAM scores for ChatGPT-4 demonstrate that it provided more suitable patient education materials.

In terms of usability, ChatGPT-4 performed better than Google on the HITS. Its user-friendly interface and more accessible presentation of information allowed patients to access health-related information more efficiently, supporting more informed decision-making.

While Google naturally scored higher on the VPI due to its broader reach, the lack of HONcode certification for many Google search results raised concerns about the reliability of the information provided. ChatGPT-4's provision of standardized and reliable information helps reduce the risk of patients encountering incorrect or misleading content. This finding underscores the importance of evaluating not only the speed and popularity of information but also its reliability.

The strong positive correlations observed between DISCERN, GQS, and JAMA scores suggest that these evaluation tools consistently measure the quality of information. This emphasizes the importance of using multiple criteria in the evaluation of medical information and provides a methodological foundation for future studies. Additionally, using these tools in combination may offer a more comprehensive and balanced assessment of AI-driven content, which could serve as a guideline for healthcare providers when directing patients to reliable sources of online information.

There are some limitations to our study. First, only 20 questions related to colon cancer were examined, so the findings may not generalize to other medical topics or a broader set of questions. Additionally, the evaluations were based on assessment tools that may contain subjective elements, and variability between different evaluators is possible. Finally, given the ongoing updates to AI models and the dynamic nature of online information sources, our results are valid only for a specific time period. Future studies should replicate this approach with a broader range of questions and different medical conditions to test the generalizability of these findings and determine the stability of AI responses over time.

## Conclusions

In conclusion, this study demonstrated that ChatGPT-4 provided more reliable, higher-quality, and user-centered information for patient questions related to colon cancer. AI-based platforms exhibit significant potential as valuable tools for delivering accurate information to both healthcare professionals and patients. Future research should expand on these findings by exploring diverse medical topics and larger, more varied populations to further define AI’s role and effectiveness in healthcare information services.

## Appendices

Chatgpt and Google answers are presented in Tables 11, 12.

**Table 11:** ChatGPT answers

ChatGPT Answers
1. What are the early symptoms of colon cancer?
Answer: The early signs of colon cancer may include changes in bowel habits (diarrhea or constipation), blood in the stool, unexplained weight loss, fatigue, and abdominal discomfort or cramps. However, many early-stage cases may not present with noticeable symptoms.
2. How is colon cancer diagnosed?
Answer: Colon cancer is diagnosed through a combination of medical history, physical examination, and diagnostic tests. These tests include colonoscopy, biopsy (tissue sampling), imaging techniques such as CT scans, and blood tests to check for cancer markers, such as carcinoembryonic antigen (CEA).
3. How does colonoscopy detect colon cancer?
Answer: A colonoscopy allows the doctor to examine the inside of the colon and rectum using a long, flexible camera tube, thereby detecting colon cancer. During the procedure, abnormal growths (polyps) can be identified, and if necessary, tissue samples can be taken to determine if cancer is present.
4. What is the role of genetic factors in colon cancer?
Answer: Genetic factors play a significant role in colon cancer. Inherited conditions such as Lynch syndrome and familial adenomatous polyposis (FAP) increase the risk of developing colon cancer. Additionally, having a family history of colon cancer can elevate an individual's risk.
5. What are the differences between colon cancer and irritable bowel syndrome (IBS)?
Answer: Colon cancer is a malignant growth in the colon, whereas IBS is a functional gastrointestinal disorder without structural abnormalities. Colon cancer presents with symptoms such as blood in the stool, unexplained weight loss, and anemia, while IBS is characterized by cramping, bloating, diarrhea, or constipation, without the presence of blood in the stool.
6. What methods are used in the treatment of colon cancer?
Answer: Treatment options for colon cancer include surgery, chemotherapy, radiation therapy, targeted therapy, and immunotherapy. The choice of treatment depends on the stage and location of the cancer, as well as the patient’s overall health condition.
7. When is surgical intervention necessary in the treatment of colon cancer?
Answer: Surgical intervention is necessary when colon cancer is localized and can be removed to prevent its spread. Surgery is often the first treatment option, especially for early-stage colon cancer. It may also be used in later stages to remove tumors or manage complications.
8. When is chemotherapy used in the treatment of colon cancer?
Answer: Chemotherapy is used when colon cancer has spread beyond the colon (metastasis) or when there is a risk of recurrence after surgery. It can be administered before surgery to shrink tumors or after surgery to eliminate any remaining cancer cells.
9. What are the newest treatment methods for colon cancer?
Answer: New treatment methods for colon cancer include targeted therapies that focus on specific cancer cells, immunotherapies that boost the body’s immune response to fight cancer, and advanced surgical techniques such as minimally invasive and robotic surgery.
10. What are the side effects of colon cancer treatment?
Answer: Side effects vary depending on the treatment but may include fatigue, nausea, vomiting, hair loss, diarrhea, neuropathy, and an increased risk of infection. Surgery may lead to pain, risk of infection, and in some cases, the need for a colostomy.
11. What is the recovery process like after colon cancer surgery?
Answer: Post-surgery recovery involves pain management, infection prevention, and gradually returning to normal activities. Patients may need to follow specific dietary guidelines, engage in light exercises, and attend follow-up appointments for monitoring.
12. What is a colostomy, and is it necessary after colon cancer surgery?
Answer: A colostomy is a surgical procedure that creates an opening in the abdomen to direct the contents of the colon into a bag outside the body. It may be necessary if part of the colon or rectum is removed during cancer surgery, but it is not required for all colon cancer surgeries.
13. What should be done and taken care of after colon cancer surgery?
Answer: After surgery, patients should focus on wound care, pain management, gradual reintroduction of food, and staying active as recommended. They should monitor for signs of complications such as fever, severe pain, or changes in bowel habits, and attend follow-up visits.
14. What is the risk of recurrence after colon cancer treatment?
Answer: The risk of recurrence depends on the stage of the cancer at diagnosis, the success of the treatment, and whether any cancer cells remain. Regular monitoring and follow-up care are essential for detecting a possible recurrence early.
15. How How should patients undergoing colon cancer treatment eat?
Answer: Patients should focus on a balanced diet rich in fruits, vegetables, whole grains, and lean proteins, while avoiding processed foods, red meat, and excessive fats. Staying hydrated and managing gastrointestinal symptoms related to treatment is also important.
16. What lifestyle changes are recommended to reduce the risk of colon cancer?
Answer: To reduce the risk of colon cancer, individuals are advised to maintain a healthy weight, stay physically active, avoid smoking and excessive alcohol consumption, and follow a diet rich in fiber and low in processed and red meat.
17. What screening tests are recommended for colon cancer prevention?
Answer: Screening tests for colon cancer include colonoscopy, fecal occult blood tests (FOBT), sigmoidoscopy, and stool DNA tests. Colonoscopy is the most comprehensive, allowing both detection and removal of polyps during the procedure.
18. Which foods increase the risk of colon cancer?
Answer: Foods that increase the risk of colon cancer include processed meats, red meat, high-fat diets, and diets low in fiber. Excessive alcohol consumption and sugary foods can also contribute to the increased risk.
19. Who is at higher risk of developing colon cancer?
Answer: Those at higher risk include individuals with a family history of colon cancer, those with inherited genetic syndromes, people over the age of 50, individuals with inflammatory bowel disease (IBD), and those who consume a diet rich in processed and red meats.
20. What support groups or resources are available for colon cancer patients?
Answer: Organizations such as the American Cancer Society, the Colon Cancer Coalition, and the Cancer Support Community offer support groups for colon cancer patients. These groups provide emotional support, educational resources, and connections with other patients and survivors.

**Table 12:** Google answers

Google Answers
1. What are the early symptoms of colon cancer?
Answer:
Symptoms
• A change in bowel habits, such as more frequent diarrhea or constipation.
• Rectal bleeding or blood in the stool.
• Ongoing discomfort in the belly area, such as cramps, gas, or pain.
• A feeling that the bowel doesn't empty all the way during a bowel movement.
• Weakness or tiredness.
• Losing weight without trying.
2. How is colon cancer diagnosed?
Answer: The main test for bowel cancer is a colonoscopy. This is where a thin, flexible, tube with a camera is used to look inside your bowel. It may be uncomfortable but it should not be painful. You'll be offered a sedative and painkillers to help make you feel more comfortable.
3. How does a colonoscopy detect colon cancer?
Answer: Colonoscopy is one of the most sensitive tests currently available for colon cancer screening. The doctor can view your entire colon and rectum. Abnormal tissue, such as polyps, and tissue samples (biopsies) can be removed through the scope during the exam.
4. What is the role of genetic factors in colon cancer?
Answer: Some gene mutations passed through generations of your family can increase your risk of colorectal cancer significantly. The most common inherited syndromes that increase colorectal cancer risk are familial adenomatous polyposis and Lynch syndrome, also known as hereditary nonpolyposis colorectal cancer.
5. What are the differences between colon cancer and irritable bowel syndrome (IBS)?
Answer: Colon Cancer: How to Tell the Difference. Irritable bowel syndrome (IBS) and colon cancer symptoms look alike. However, unlike IBS, colon cancer may cause bloody stool, fatigue, and unexplained weight loss, among other unique symptoms. IBS is a chronic disorder of your large intestine.
6. What methods are used in the treatment of colon cancer?
Answer: Colon cancer treatment usually involves surgery to remove the cancer. Your health care team might recommend other treatments, such as radiation therapy and chemotherapy. Your treatment options depend on the cancer's location and its stage
7. When is surgical intervention necessary in the treatment of colon cancer?
Answer: Stages 1 to 3: In general, surgery is considered the best choice for colon cancers stages 1 to 3: Stage 1 and 2 cancer is contained to the wall of the colon and possibly nearby tissue. Stage 3 cancer has spread from the colon to nearby lymph nodes, but not to other parts of the body.
8. When is chemotherapy used in the treatment of colon cancer?
Answer: Chemo may be used at different times during treatment for colorectal cancer: Neoadjuvant chemo is given (sometimes with radiation) before surgery to try to shrink the cancer and make it easier to remove. This is often done for rectal cancer. Adjuvant chemo is given after surgery.
9. What are the newest treatment methods for colon cancer?
Answer: Treatment for Colorectal Cancer. Surgically removing the cancer is the most common treatment for many stages of colorectal cancer. Chemotherapy, radiation, targeted therapy, radiofrequency ablation, and cryosurgery are other treatments that may be used to treat colorectal cancer, depending on the stage.
10. What are the side effects of colon cancer treatment?
Answer: The side effects include the following:
• Dehydration: Dehydration means your body does not have enough fluids, and this can be dangerous. ...
• Skin rash and discomfort. Treatment may make your skin bumpy, red, itchy, or irritated.
• Nausea and vomiting.
• Diarrhea.
• Fatigue.
• Peripheral neuropathy.
• Mucositis.
• Hand and foot syndrome.
11. What is the recovery process like after colon cancer surgery?
Answer: Your recovery time after the operation will depend on your age, whether you had open or keyhole surgery, whether you have a stoma, and your general health. You will probably be in hospital for 2-7 days, but it can take 2-3 months to fully recover.
12. What is a colostomy, and is it necessary after colon cancer surgery?
Answer: A colostomy is surgery to create an opening for the colon (large intestine) through the belly (abdomen). A colostomy may be short-term (temporary) or long-term (permanent). It's often done after bowel surgery or injury. Most permanent colostomies are end colostomies.
13. What should be done and taken care of after colon cancer surgery?
Answer: Right after surgery, you will need to be on a clear liquid diet. This diet continues for the rest of that day. The day after surgery, you will get back to a regular diet. A dietitian will be available to help you while you're in the hospital.
14. What is the risk of recurrence after colon cancer treatment?
Answer: The rate of colorectal cancer survival five years after initial diagnosis has been on the rise. But with survival comes a new risk - that of recurrence. Colorectal cancer recurrence within five years after treatment ends is in the range of 7 to 42 percent, depending on the stage of the cancer.
15. How should patients undergoing colon cancer treatment eat?
Answer: After surgery, you will be on a “low residue” diet. This is a diet low in fiber. It will minimize the amount of work for your colon and rectum. Think “white bread and white rice, not whole grain bread and brown rice,” and avoid foods like raw vegetables, nuts and seeds, whole grain products, beans, and fried meats.
16. What lifestyle changes are recommended to reduce the risk of colon cancer?
Answer: Some studies suggest that people may reduce their risk of developing colorectal cancer by: Increasing physical activity. Keeping a healthy weight. Eating a diet low in animal fats and high in fruits, vegetables, and whole grains
17. What screening tests are recommended for colon cancer prevention?
Answer: Everyone ages 45 to 75 years needs to get screened for colorectal cancer. Screening saves lives because it can help catch cancer early, when it's easier to treat. There are 3 main types of colorectal cancer screening tests: colonoscopy, flexible sigmoidoscopy, and stool tests.
18. What foods increase the risk of colon cancer?
Answer: Some foods to avoid to lower your risk of developing colorectal cancer include:
• Red meat.
• Processed meats.
• White bread.
• Sugary beverages.
19. Who is at higher risk of developing colon cancer?
Answer: If you are overweight or obese (very overweight), your risk of developing and dying from colorectal cancer is higher. Being overweight raises the risk of colorectal cancer in people, but the link seems to be stronger in men. Getting to and staying at a healthy weight may help lower your risk
20. What support groups or resources are available for colon cancer patients?
Answer:
• Get screened.
• Maintain a healthy weight.
• Don't smoke.
• Be physically active.
• Limit alcohol: zero is best.
• Limit red meat, especially processed meat.
• Get enough calcium and vitamin D.
• Eat more whole grains and fiber.
